# Resting-state brain oscillations predict cognitive function in psychiatric disorders: A transdiagnostic machine learning approach

**DOI:** 10.1016/j.nicl.2021.102617

**Published:** 2021-03-19

**Authors:** Kaia Sargent, UnYoung Chavez-Baldini, Sarah L. Master, Karin J.H. Verweij, Anja Lok, Arjen L. Sutterland, Nienke C. Vulink, Damiaan Denys, Dirk J.A. Smit, Dorien H. Nieman

**Affiliations:** a Amsterdam University Medical Centers (location AMC), University of Amsterdam, Department of Psychiatry, Meibergdreef 9, Amsterdam, Netherlands; b Max Planck Institute for Biological Cybernetics, Max Planck-Ring 8, 72076 Tuebingen, Germany

**Keywords:** Resting-state EEG, Transdiagnostic psychiatry, Machine learning, Cognitive function

## Abstract

**Background:**

Cognitive dysfunction is widespread in psychiatric disorders and can significantly impact quality of life. Deficits cut across traditional diagnostic boundaries, necessitating new approaches to understand how cognitive function relates to large-scale brain activity and psychiatric symptoms across the diagnostic spectrum.

**Objective:**

Using random forest regression, we aimed to identify transdiagnostic patterns linking cognitive function to resting-state EEG oscillations.

**Methods:**

216 participants recruited through an outpatient psychiatric clinic completed the Cambridge Neuropsychological Test Automated Battery and underwent a 5-minute eyes-closed resting state EEG recording. We built random forest regression models to predict performance on each cognitive test using the resting-state EEG power spectrum as input, and we compared model performance to a sampling distribution constructed with random permutations. For models that performed significantly better than chance, we used feature importance estimates to identify features of the EEG power spectrum that are predictive of cognitive functioning.

**Results:**

Random forest models successfully predicted performance on measures of episodic memory and associative learning (Paired Associates Learning, PAL), information processing speed (Choice Reaction Time, CRT), and attentional set-shifting and executive function (Intra-Extra Dimensional Set Shift, IED). Oscillatory power in the upper alpha range was associated with better performance on PAL and CRT, while low alpha power was associated with worse CRT performance. Beta power predicted poor performance on all three tests. Theta power was associated with good performance on PAL, and delta and theta oscillations were identified as predictors of good performance on IED. No differences in cognitive performance were found between diagnostic categories.

**Conclusion:**

Resting oscillations are predictive of certain dimensions of cognitive function across various psychiatric disorders. These findings may inform treatment development to improve cognition.

## Highlights

• Resting EEG activity associated with cognitive function in psychiatric disorders.
• Using EEG, random forest modeling predicts cognitive performance but not diagnosis.
• High alpha oscillations associated with better episodic memory and processing speed.
• Beta oscillations associated with worse performance in several cognitive domains.
• EEG power changes in psychiatric disorders may be related to cognitive dysfunction.

## 1 Introduction

While psychiatric research and treatment have traditionally focused on the affective changes that characterize mental disorders, cognitive function is gaining increasing attention as a relevant dimension of psychiatric illness. Cognitive deficits are widespread across disorders and can significantly impact overall functioning and quality of life (Millan et al., 2012). As such, cognitive difficulties are often a primary complaint for which patients seek treatment (Nieman, 2016). Understanding the neurobiological mechanisms underlying cognitive function should therefore be a central goal in efforts to improve treatment and enhance quality of life for patients.

Cognitive function encompasses a broad range of domains including memory, attention, language, problem solving and decision making. Every major diagnostic category in the Diagnostic and Statistical Manual of Mental Disorders (DSM) is associated with altered cognitive functioning in at least one domain (Millan et al., 2012). Perturbations in executive function occur across multiple disorders, affecting various subdomains including planning and decision making (e.g. depressive disorders; Marazziti et al., 2010), cognitive flexibility (e.g. autism spectrum disorder (ASD); Robinson et al., 2009), and inhibitory control (e.g. obsessive–compulsive disorder (OCD); Penades et al., 2007) and bipolar disorder (Kurtz and Gerraty, 2009)). Executive function is most severely compromised in individuals with schizophrenia, who have deficits in all of these subdomains (Dickinson and Harvey, 2009, Kalkstein et al., 2010). Deficits in working memory and semantic memory are also common in schizophrenia (Barnett et al., 2010), while episodic memory is compromised in depression, bipolar disorder, schizophrenia, ASD, posttraumatic stress disorder (PTSD) and OCD (Millan et al., 2012). Changes in attention are also associated with most disorders, including attention deficit hyperactivity disorder (ADHD; Vaidya and Stollstorff, 2008), PTSD (McNally, 2006), OCD (Burdick et al., 2008), and generalized anxiety disorder (GAD; Castaneda et al., 2008).

It is difficult to disentangle disorder-specific patterns of cognitive impairment from general deficits transcending diagnostic categories. Cognitive function is highly complex, involving multiple cognitive domains that interact with each other and with emotional and social processing, making it difficult to isolate individual cognitive processes for scientific study. Furthermore, psychiatric disorders themselves are not clearly delineated but instead have broad symptom overlap and high rates of comorbidity. To address this issue, recent research initiatives have attempted to elucidate biological processes underlying psychopathology more broadly without being bound to traditional diagnostic categories (e.g., Research Domain Criteria (RDoc; (Insel et al., 2010); Hierarchical Taxonomy of Psychopathology (HiTOP; Kotov et al., 2017)). Such transdiagnostic approaches focus on specific domains such as cognition, arousal, and emotion regulation, which are implicated in psychopathology across the diagnostic spectrum and are more closely linked to basic biological processes than complex and heterogeneous psychiatric disorders are. By taking a fine-grained, bottom-up approach to link specific dimensions of functioning with biological parameters, we may develop a more biologically grounded understanding of psychiatric illness across the diagnostic spectrum.

Cognitive function can be understood in biological terms as precisely orchestrated interactions of brain regions and networks. Breakdowns in the coordination of brain activity are also associated with diverse forms of psychopathology, as evidenced by aberrant network organization in all major psychiatric disorders (Buckholtz and Meyer-Lindenberg, 2012). Measuring large-scale neural activity can therefore provide important insights into brain function subserving cognition and implicated in psychopathology.

Electroencephalography (EEG) provides millisecond temporal resolution in measuring ongoing electrical activity, which constitutes the basis of information exchange across the brain in the form of synchronized oscillations (Basar et al., 2001). These oscillations are evident in the EEG power spectrum, particularly in specific frequency ranges referred to as alpha (8–13 Hz), beta (13–30 Hz), gamma (30–90 Hz), theta (4–8 Hz), and delta (1–4 Hz). A significant proportion of the EEG literature in psychiatric illness focuses on oscillatory power in these frequency bands, with the EEG signals typically recorded in the resting-state. The changes observed between psychiatric cases and controls are, however, highly variable within disorders and not diagnostically specific (for review see Newson and Thiagarajan, 2018). Resting-state EEG power changes likely hold important information about brain function in psychiatric illness, but it is unclear what specific pathophysiological processes such changes reflect. Thus, taking a more fine-grained approach to link specific EEG changes to specific domains of functioning may offer new interpretations of neurophysiological abnormalities in psychiatric disorders.

This study takes a transdiagnostic approach to explore the relationship between resting-state EEG activity and cognitive function across multiple psychiatric disorders. 216 participants with disorders across seven diagnostic categories were recruited from an outpatient psychiatric clinic and included in the study. Machine learning methods are becoming increasingly employed to identify novel patterns in large datasets, and are particularly well suited for the emerging field of transdiagnostic computational psychiatry to mine information-rich biological signals such as EEG and identify relationships with symptom domains across disorders. One study of patients with schizophrenia employed machine learning methods to identify task-based EEG features that predict working memory performance (Johannesen et al., 2016), highlighting the utility of machine learning methods for extracting relevant features from high-dimensional EEG data. The present study employs a similar approach, using random forest regression to identify resting-state EEG features associated with cognitive performance across multiple cognitive domains and psychiatric disorders. This approach may shed light on the neurobiological basis and clinical relevance of cognitive dysfunction in psychiatric illness.

## 2 Materials and methods

### 2.1 Sample

Data are reported from 216 participants who were recruited through an outpatient clinic of the Department of Psychiatry at the Amsterdam University Medical Centers (UMC) in Amsterdam, the Netherlands. Inclusion criteria were: age 18–75 years, ability to give informed consent, having a *DSM-IV-TR* or *DSM-V* diagnosis, being clinically stable, and being fluent in Dutch. Exclusion criteria were: high risk of suicide, unstable medical disorder, premorbid IQ < 70, history of seizures or neurological disorder. Informed consent was obtained from all participants. Of 955 patients who participated in the Across study, 256 participants agreed to participate in the EEG substudy. 6 participants were excluded because their EEG data were unusable due to technical issues. Of the remaining participants, 216 completed the CANTAB battery and were included in analyses.

### 2.2 Procedure

The Across study is an ongoing, observational longitudinal cohort study and consists of the assessment of cognitive performance, psychiatric symptoms, and collection of biological data (DOI https://doi.org//10.17605/OSF.IO/YHVTB). All instruments and procedures are described in Nieman et al. (2020). Participants underwent an extensive psychiatric and medical assessment at the outpatient clinic, performed by experienced psychiatrists and psychologists. The current study uses baseline data from the computerized cognitive assessment, EEG recordings, and symptomatology questionnaires (see Supplementary Material). The study protocol was approved by the Medical Ethical Review Committee of the Amsterdam UMC (ABR no. NL55751.018.15), and data is stored according to privacy laws.

#### 2.2.1 Cognitive assessment

Cognitive functioning was assessed with the Cambridge Neuropsychological Test Automated Battery (CANTAB Cognitive Assessment Software, 2018). The CANTAB test battery is composed of the following subtests: Verbal Recognition Memory (VRM), Rapid Visual Information Processing (RVP), Intra/Extradimensional Set Shift (IED), Choice Reaction Time (CRT), One Touch Stockings of Cambridge (OTS), Paired Associates Learning (PAL), and Spatial Working Memory (SWM). Descriptions of the subtests are found in Table 1.

**Table 1:** Description of Cambridge Neuropsychological Test Automated Battery subtests.

Subtest	Description
Verbal Recognition Memory (VRM)	Assesses free recall, and immediate and delayed recognition memory for verbal information
Rapid Visual Information Processing (RVP)	Tests visual sustained attention and processing speed
Intra/ Extradimensional Set Shift (IED)	Assesses rule acquisition and attentional set shifting
Choice reaction time (CRT)	Measures alertness and motor speed
One Touch Stockings of Cambridge (OTS)	A planning test measuring frontal lobe functioning
Paired Associates Learning (PAL)	Assesses visual episodic memory and learning
Spatial Working Memory (SWM)	Assesses working memory and strategy use

#### 2.2.2 EEG acquisition and processing

EEG was recorded with a WaveGuard cap with Ag/AgCl electrodes with standard 10/10 layout fed into the 64-channel ANT TMSi Refa amplifier, using Fpz as ground, horizontal EOG electrodes affixed to the outer canthus and vertical EOG electrodes affixed above and below the right eye, and two mastoid channels (M1/M2). The vertex electrode (Cz) was used as the recording reference. Eyes-closed resting state EEG was recorded for 5 min, in addition to eyes-open resting state and an auditory oddball task for a total recording session of 45 min. Eyes-closed resting state was used for the current analysis, since the majority of studies of EEG oscillations in psychiatric disorders report data from eyes-closed recording (Newson and Thiagarajan, 2018). Recordings were sampled at 512 Hz with a 128 Hz low-pass filter.

All analyses were performed in MATLAB R2018b. EEG preprocessing was performed for each subject using EEGLAB (Delorme and Makeig, 2004). Data were re-referenced offline to an average reference and filtered using a FIR bandpass filter from 1 to 50 Hz. Bad channels were removed and interpolated. Data were epoched into 2-second segments to manually reject artifactual epochs, after which data were re-concatenated into continuous data. Independent component analysis was performed to manually identify and reject noise components. Ocular and muscular artifacts were removed using blind source separation and canonical correlation analysis techniques implemented in the AAR plugin for EEGLAB (De Clercq et al., 2006).

For all 64 channels, a fast Fourier transform was computed after applying a 512-point Hanning window. The resulting power spectrum was segmented into bins of 1 Hz, ranging from 1 to 50 Hz (50 bins) and log-transformed. To control for the effects of age and gender, power spectra for all electrodes were regressed on age and gender, and residual scores were used as corrected power values for all analyses.

#### 2.2.3 Random forest prediction

To estimate performance on each CANTAB cognitive test, we used a random forest model with power at each channel in 1-Hz bins from 1 to 50 Hz as input features after correcting for age and gender. Random forest is one of the most popular machine learning algorithms for classification and regression (Breiman, 2001), and is particularly suited for high dimensional data. The algorithm creates a large number of decision trees, where each tree is trained on a bootstrap sampling of the data with a random subsample of features. The algorithm builds a maximally informative decision tree for each sampling, with each level moving into a branch based on a critical value for an input feature. After multiple decisions, each tree leads to end nodes that are associated with a classification into either of two groups. In the regression extension, each decision tree assigns a value for the outcome variable based on decisions made for the sampled features falling above or below a certain threshold. After all decision trees are run and the forest of trees is created, the prediction of the random forest is the average prediction of the individual trees. Bootstrap aggregation or “bagging” of decision trees means that each tree is trained on a subsample of the data, so the performance of each model on its left-out samples (“out-of-bag” observations) when averaged provides an estimate of model accuracy. In this way, out-of-bag performance provides a metric of generalization performance that is very similar to cross-validation.

Because our primary aim was to identify EEG features associated with cognitive performance, our choice of random forest regression among various machine learning techniques was primarily due to its utility in estimating feature importance. Besides being versatile in prediction of continuous and categorical outcomes, random forest naturally allows for the inspection of predictor importance. Due to its randomization and 'bagging' component, it will result in a gradual distribution of importance of features, in contrast with, for example, penalized regressions that will result in sparse weights across space and frequency, especially when features are highly correlated. This is crucial for visualizing scalp topography of predictor importance.

For each cognitive test, we built a random forest with the number of trees set to 20,000 and with the number of predictors to sample set to 12. These hyperparameters were selected not to optimize model performance *per se*, but rather to optimize predictor importance estimates to determine which EEG features are most predictive of cognitive functioning. We found that predictor importance estimates were highly variable with <5000 trees, and predictor importance estimates became steadily less variable as the number of trees was increased to 20,000. Model performance did not improve above 5000 trees, and neither model performance nor predictor importance estimates improved with >12 predictors sampled.

Model performance was evaluated using the coefficient of determination or Nash-Sutcliffe efficiency (NSE), reflecting model fit between observed cognitive scores and predicted scores for out-of-bag observations. NSE is computed as $\mathrm{NSE}=1-\frac{\sum {\left(Y_{m}-Y_{p}\right)}^{2}}{\sum {\left(Y_{m-}\overset{-}{Y}\right)}^{2}}$, where *Y_m_* and *Y_p_* are observed and predicted values, respectively, and y is the mean of observations. NSE ranges from –∝ to 1.

For each model, permutation testing was conducted to determine if the model performed significantly better than chance. Cognitive test scores were randomly permuted and a random forest model was built for each permutation, thus obtaining a null distribution of the NSE statistic. The p-value is the frequency of random models that perform equal to or better than the original model, reflecting the probability of obtaining equal or better model performance due to chance alone. The significance threshold was set at p = 0.00714 (0.05/7, correcting for number of cognitive tests), and 2000 permutations were performed to provide a p-value resolution of 0.0005 (1/2000 = 0.0005).

For models that performed significantly better than chance, predictor importance was estimated for EEG features as the increase in prediction error if the values of the predictor are randomly permuted for out-of-bag observations. To identify frequencies with the highest predictor importance for each model, we plotted predictor importance by frequency (averaged across electrodes) and selected peaks, defined as local maxima exceeding one standard deviation above the mean predictor importance estimate. To investigate the direction of effect for each frequency predictor on cognitive outcome, we identified the top three channels with greatest predictor importance within each selected frequency and examined their relationships with cognitive performance using univariate regression models.

#### 2.2.4 Statistical analysis

Follow-up analyses were performed to assess differences among diagnostic groups. For cognitive tests that were found to be predictable from EEG data (based on significance of permutation testing), a Welch’s ANOVA was performed to determine if cognitive scores differed among diagnostic groups. Welch’s ANOVA was used due to unequal variances across groups. We first corrected the scores by residualizing them for age and gender across the whole sample as in the random forest models. Next, the Welch’s ANOVA included diagnostic groups with n > 10 (see Table 2). Further analyses to investigate possible effects of medication and diagnostic category on predictor-outcome relationships are included in Supplementary Material (Supplementary Tables 2 and 3). An important and clinically relevant question is how cognitive (dys)function interacts with other dimensions of psychiatric illness. We decided to investigate the relationship between cognitive function and other symptom dimensions in order to more effectively interpret the relationship between EEG activity and cognition. We therefore performed a series of Pearson correlations between CANTAB scores and symptom scores on five symptom dimensions. These symptom dimensions were identified using factor analysis of self-report symptom questionnaires (see Supplementary Material) and were labeled as social/interpersonal, anxious, depressive, somatic, and anomalous (psychosis-spectrum symptoms). To rule out the possibility that predictability of cognitive performance is related to specific diagnosis, we used the same model parameters to build random forest models to predict each diagnosis and assessed performance using the same methods described above.

**Table 2:** Participant demographics and CANTAB scores.

	Total	MDD	BP	PSY	OCD	GAD	ASD	ID-NOS
**n**	216	34	4	15	49	9	4	101
**Age** mean (SD)	38.6 (15.0)	41.5 (15.7)	38.0 (18.3)	29.3 (10.6)	43.7 (14.8)	45.1 (16.3)	26.3 (6.3)	36.5 (14.4)
**Gender** # male (%)	83 (38.4)	14 (41.2)	2 (50.0)	8 (53.3)	21 (42.9)	4 (44.4)	3 (75.0)	31 (30.7)
**Medication status** # medicated (%)	68 (31.5)	16 (47.1)	1 (25.0)	9 (60.0)	14 (28.6)	4 (44.4)	0 (0.0)	24 (23.8)
**Premorbid IQ** mean (SD)	103.1 (13.1)	102.9 (15.2)	100.5 (13.2)	105.8 (16.0)	106.1 (14.6)	95.7 (11.7)	99.5 (1.73)	102.2 (11.3)
**CRT** mean (SD)	351 (87.9)	395 (1 1 8)	337 (61.9)	305 (41.8)	351 (63.8)	393 (2 2 1)	322 (40.0)	340 (66.9)
**IED** mean (SD)	26.4 (27.4)	29.7 (40.0)	21.8 (23.0)	30.4 (26.9)	30.8 (31.0)	39.2 (38.8)	9.50 (2.65)	22.2 (18.1)
**OTS** mean (SD)	1.39 (0.255)	1.39 (0.270)	1.42 (0.373)	1.35 (0.202)	1.45 (0.281)	1.58 (0.384)	1.49 (0.212)	1.34 (0.219)
**PAL** mean (SD)	12.3 (18.9)	18.1 (26.6)	11.3 (13.7)	10.2 (11.5)	11.9 (13.0)	24.0 (29.4)	9.50 (10.1)	9.87 (18.0)
**RVP** mean (SD)	0.898 (0.054)	0.876 (0.068)	0.870 (0.066)	0.884 (0.053)	0.903 (0.042)	0.868 (0.053)	0.952 (0.005)	0.908 (0.050)
**SWM** mean (SD)	21.0 (18.5)	24.9 (20.4)	27.3 (12.1)	27.9 (25.7)	25.3 (20.9)	28.0 (18.4)	9.25 (13.3)	16.2 (14.3)
**VRM** mean (SD)	6.75 (2.70)	6.29 (2.60)	9.25 (3.59)	6.13 (2.45)	7.00 (3.19)	5.78 (2.17)	5.25 (2.22)	6.92 (2.49)

*Note:* MDD = Major depressive disorder; BP = Bipolar disorder; PSY = Psychosis spectrum disorders; OCD = Obsessive-compulsive disorder; GAD = Generalized anxiety disorder; ASD = Autism spectrum disorder; ID-NOS = Impulse-control disorder, not otherwise specified (misophonia). Note: large ID-NOS sample is due to specialized misophonia research group located at the AUMC. Premorbid IQ assessed with National Adult Reading Test (NART; Nelson and Willison, 1991). CRT = Choice Reaction Time, mean correct latency; IED = Intra-Extra Dimensional Set Shift, total errors adjusted; OTS = One Touch Stockings of Cambridge, mean choices to correct; PAL = Paired Associates Learning, total errors adjusted; RVP = Rapid Visual Information Processing, A-prime; SWM = Spatial Working Memory, between errors; VRM = Verbal Recognition Memory, free recall total correct.

## 3 Results

Table 2 shows demographic information and CANTAB cognitive test scores for 216 participants. Further medication data are presented in Supplementary Table 1.

Table 3 shows random forest model performance statistics for each cognitive test. NSE (ranging from –∞ to 1) reflects model fit between observed scores and predicted scores for out-of-bag observations. The p-value is the frequency of random permutation models that perform equal to or better than the original model, reflecting the probability of obtaining equal or better model performance due to chance alone. For models that performed significantly better than chance, predictor importance was estimated for EEG features.

**Table 3:** Random forest model performance for each cognitive test

	NSE	p-value*
VRM	−0.0018	0.0560
RVP	−0.0487	0.5605
IED	0.0226	**0.0070**
CRT	0.0242	**0.0065**
OTS	−0.0419	0.4500
PAL	0.0348	**0.0055**
SWM	−0.0434	0.4805

*Significant p-values (<0.00714) shown in bold

### 3.1 Paired associate learning

**Fig. 1:**
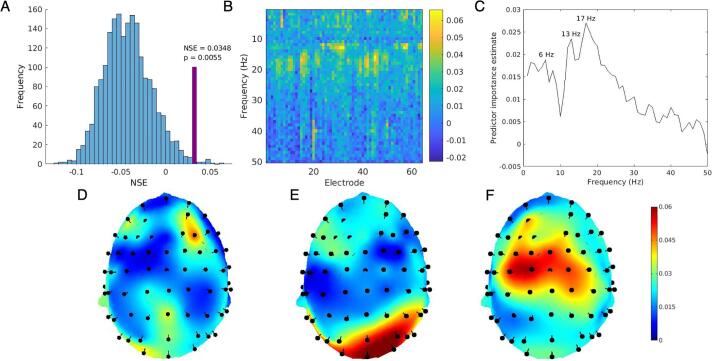
(A) Distribution of NSE values from 2000 permutations of random forest model with PAL scores randomly shuffled. Observed NSE shown in purple. 11 out of 2000 permutations exceeded the observed NSE value, yielding a p-value of 0.0055 (B) PAL predictor importance estimates for all 3200 predictors (electrode × frequency). (C) Estimated predictor importance by frequency (averaged across electrodes). (D) Topography of predictor importance at 6 Hz (corresponding to predictor importance peak at 6 Hz, see C). (E) Topography of predictor importance at 13 Hz. (F) Topography of predictor importance at 17 Hz. (For interpretation of the references to colour in this figure legend, the reader is referred to the web version of this article.)

The random forest model performed significantly better than chance predicting PAL score from EEG data. Fig. 1A shows the observed NSE value relative to the null distribution of permuted NSE statistics. Fig. 1B–F show predictor importance estimates, computed for each predictor as the increase in prediction error if the values of the predictor are randomly permuted for out-of-bag observations. Topography of predictor importance estimates for each frequency (1–50 Hz in 1 Hz bins) are shown in Supplementary Material Fig. 1. Fig. 1D–F show a selection of topographical maps corresponding to frequency peaks, identified at 6 Hz, 13 Hz, and 17 Hz. These peaks were selected for subsequent analyses to determine directions of effects.

To assess the direction of effect for frequencies with high predictor importance, linear regression models were used with power as independent variables and PAL score (total errors adjusted) as the dependent variable. For each peak frequency, three channels with the highest predictor importance were tested in univariate models. 6 Hz power at F4, AF4 and O1 all exhibited inverse relationships with PAL errors (greater power associated with better performance; β_F4_ = −0.49, β_AF4_ = −0.41, β_O1_ = −0.25). 13 Hz power also showed an inverse relationship with PAL errors (β_Oz_ = −0.27, β_O2_ = −0.29, β_PO4_ = −0.30). 17 Hz power exhibited a positive relationship with PAL errors (greater power associated with worse performance; β_C1_ = 0.48, β_C3_ = 0.49, β_FCz_ = 0.52).

### 3.2 Choice Reaction Time

**Fig. 2:**
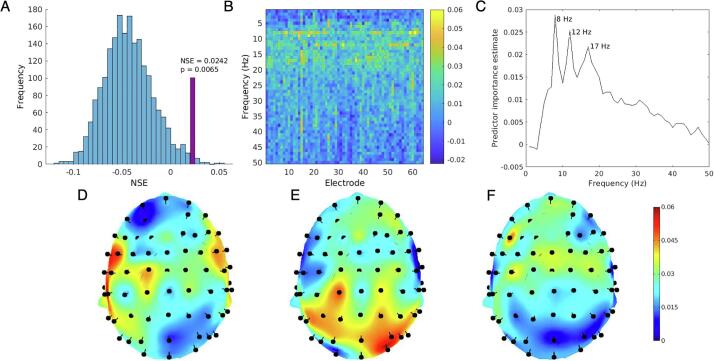
(A) Distribution of NSE values from 2000 permutations of random forest model with CRT scores randomly shuffled. Observed NSE shown in purple. 13 out of 2000 permutations exceeded the observed NSE value, yielding a p-value of 0.0065 (B) CRT predictor importance estimates for all 3200 predictors (electrode × frequency). (C) Estimated predictor importance by frequency (averaged across electrodes). (D) Topography of predictor importance at 8 Hz (corresponding to predictor importance peak at 8 Hz, see C). (E) Topography of predictor importance at 12 Hz. (F) Topography of predictor importance at 17 Hz. (For interpretation of the references to colour in this figure legend, the reader is referred to the web version of this article.)

The random forest model performed significantly better than chance predicting CRT scores from EEG data. Fig. 1A shows the observed NSE value relative to the null distribution of permuted NSE statistics. Fig. 2B–F show predictor importance estimates. Topography of predictor importance estimates for each frequency (1–50 Hz in 1 Hz bins) are shown in Supplementary Material Fig. 2. Fig. 2D–F show a selection of topographical maps corresponding to frequency peaks, identified at 8 Hz, 12 Hz, and 17 Hz. These peaks were selected for subsequent analyses to determine directions of effects.

To assess the direction of effect for frequencies with high predictor importance, linear regression models were used with power as independent variables and CRT score (mean correct latency) as the dependent variable. For each peak frequency, three channels with the highest predictor importance were tested in univariate models. 8 Hz power at FC5, FT7, and TP7 all exhibited positive relationships with CRT latency (greater power associated with worse performance; β_FC5_ = 0.30, β_Ft7_ = 0.39, β_TP7_ = 0.32). 17 Hz power also exhibited a positive relationship with CRT latency (β_C1_ = 0.47, β_C3_ = 0.56, β_FCz_ = 0.41). 12 Hz power exhibited an inverse relationship with CRT latency (greater power associated with better performance; β_CP1_ = −0.07, β_O2_ = −0.20, β_PO6_ = −0.21).

### 3.3 Intra-Extra dimensional set Shift

**Fig. 3:**
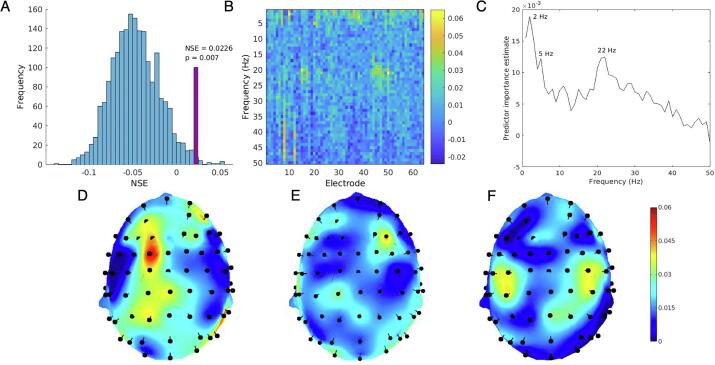
(A) Distribution of NSE values from 2000 permutations of random forest model with IED scores randomly shuffled. Observed NSE shown in purple. 14 out of 2000 permutations exceeded the observed NSE value, yielding a p-value of 0.007 (B) IED predictor importance estimates for all 3200 predictors (electrode × frequency). (C) Estimated predictor importance by frequency (averaged across electrodes). (D) Topography of predictor importance at 2 Hz (corresponding to predictor importance peak at 2 Hz, see C). (E) Topography of predictor importance at 5 Hz. (F) Topography of predictor importance at 22 Hz. (For interpretation of the references to colour in this figure legend, the reader is referred to the web version of this article.)

The random forest model performed significantly better than chance predicting IED score from EEG data. Fig. 3A shows the observed NSE value relative to the null distribution of permuted NSE statistics. Fig. 3B–F show predictor importance estimates. Topography of predictor importance estimates for each frequency (1–50 Hz in 1 Hz bins) are shown in Supplementary Material Fig. 3. Fig. 3D–F show a selection of topographical maps corresponding to frequency peaks, identified at 2 Hz, 5 Hz, and 22 Hz. These peaks were selected for subsequent analyses to determine directions of effects.

To assess the direction of effect for frequencies with high predictor importance, linear regression models were used with power as independent variables and IED score (total errors adjusted) as the dependent variable. For each peak frequency, three channels with the highest predictor importance were tested in univariate models. 2 Hz power at F1, FC1 and P8 all exhibited inverse relationships with IED errors (greater power associated with better performance; β_F1_ = −0.35, β_FC1_ = −0.50, β_P8_ = −0.44). 5 Hz power also showed an inverse relationship with IED errors (β_F4_ = −0.47, β_CP1_ = −0.38, β_CP5_ = −0.38). 22 Hz power exhibited a positive relationship with IED errors (greater power associated with worse performance; β_C4_ = 0.34, β_CP3_ = 0.13, β_CP4_ = 0.19).

### 3.4 Follow-up analyses: Clinical relationships

To assess whether PAL, CRT, and IED scores differed among diagnostic groups, we performed a Welch’s ANOVA for each cognitive test controlling for age and gender, including only diagnostic groups with n > 10 (MDD, PSY, OCD, and ID-NOS). For all three cognitive tests, scores did not differ significantly among diagnostic groups (PAL: F(3,53.14) = 1.27, p = 0.30; CRT: F(3,55.17) = 2.45, p = 0.07; IED: F(3,46.32) = 1.09, p = 0.36). There were no significant correlations between CANTAB scores and symptom scores for any of the five symptom dimensions, as seen in Table 4.

**Table 4:** Correlations between CANTAB scores and symptom dimension scores

	PAL		CRT		IED	
	r	p	r	p	r	p
Social/interpersonal	−0.128	0.080	0.138	0.058	−0.012	0.869
Anxious	−0.075	0.305	−0.073	0.352	−0.060	0.411
Depressive	−0.040	0.585	−0.080	0.271	0.029	0.691
Somatic	0.092	0.207	0.113	0.121	0.071	0.333
Anomalous	−0.048	0.515	−0.001	0.990	−0.084	0.252

We next tested whether a random forest model would also successfully predict diagnosis, which could suggest that the relationship between EEG oscillations and cognition is secondary to the relationship between EEG oscillations and specific psychopathology. We built a random forest model to predict each diagnosis, with the same model parameters that were used to predict cognitive scores. Although a classification model is generally used to predict a binary target variable (diagnosis or no diagnosis), we used a regression model to maintain methodological consistency with the continuous prediction of cognitive scores and to allow graded (continuous) prediction values, perhaps reflecting disorder severity. Table 5 shows NSE and p-values for each model. No model performed better than chance in predicting diagnosis.

**Table 5:** Random forest model performance for diagnostic categories

Diagnosis	NSE	**p-**value
MDD	−0.0299	0.2753
BP	−0.0158	0.1325
PSY	−0.0470	0.5345
OCD	−0.0355	0.3545
GAD	−0.0097	0.0950
ID-NOS	−0.0362	0.3630

## 4 Discussion

The aim of this study was to identify correlates of cognitive function in the resting EEG power spectrum, across various psychiatric disorders and multiple cognitive domains. Using resting EEG data as input, random forest models performed significantly better than chance in predicting performance in tasks measuring episodic memory and associative learning (PAL), information processing speed (CRT), and attentional set-shifting and executive function (IED). Power in the upper alpha range (12–13 Hz) was associated with better performance on PAL and CRT, while power in the beta frequency range was associated with poorer performance on all three tests. Theta oscillations were associated with better performance on PAL, and theta and delta oscillations were associated with better IED performance. Random forest models with the same hyperparameters were unable to predict diagnosis at a level above chance. Scores for PAL, CRT, and IED did not differ significantly among diagnostic groups.

### 4.1 Resting oscillations and cognition

#### 4.1.1 Alpha oscillations

We found that better performance on PAL and CRT was associated with greater power in the upper alpha range, while increased power in the lower alpha range was associated with worse CRT performance. Resting alpha power, particularly in the high-alpha range, has been found to correlate with cognitive performance and memory in particular (Klimesch et al., 1997; Mahjoory et al., 2019, Prat et al., 2016, Vogt et al., 1998). Several studies also provide evidence that individual alpha frequency (IAF) is an indicator for speed of cognitive processes (Klimesch et al., 1997; Surwillo, 1963, Surwillo, 1964), which may explain why power in the low alpha range was associated with worse CRT performance.

Alpha oscillations have been traditionally thought to reflect idling or inhibition of task-irrelevant cortical areas, given that they desynchronize in response to most task demands. However, recent findings that alpha oscillations increase in certain conditions, particularly in working memory tasks (Palva et al., 2010, Smit et al., 2009), have led to a revised understanding that ascribes alpha oscillations an active role in cognitive processing. Alpha oscillations serve a critical top-down modulatory role by acting as a selective inhibitory filter (Klimesch et al., 2007) and by controlling rhythmic changes in neural excitability (Mathewson et al., 2012, Sadaghiani and Kleinschmidt, 2016). This enables the precise timing of neuronal firing rates as a function of alpha phase and modulates higher frequency oscillations (Palva and Palva, 2007). High resting alpha power may therefore reflect effective top-down cognitive control that allows for efficient information processing (Klimesch et al., 2007). This function may be compromised in psychiatric illness, as alpha oscillations are reduced across multiple disorders (see Newson and Thiagarajan, 2018 for review). Given the critical role of alpha oscillations in modulating and maintaining global brain dynamics, further work should attempt to clarify whether reduced alpha oscillations in psychiatric disorders relate specifically to cognitive dysfunction, or if alpha reductions underlie broad network disruptions leading to diverse symptoms.

#### 4.1.2 Beta oscillations

We found that resting beta power was associated with poor performance on PAL, CRT, and IED. While beta oscillations are classically considered to be related to sensorimotor functions through the maintenance of steady muscle contractions (Baker, 2007), work in the last few decades suggests that beta oscillations play a parallel role in cognition through the maintenance of ongoing cognitive operations (Buschman and Miller, 2007). Engel and Fries (2010) offer a unifying hypothesis of beta oscillations serving to maintain the current sensorimotor and cognitive set, or “status quo”. Engel and Fries predict that pathological enhancement of beta activity is likely to result in deterioration of flexible cognitive control and efficient information processing. This could explain why elevated resting beta is associated with poorer performance on flexible set shifting (IED) and slower processing of novel stimuli (CRT). Beta oscillations may maintain the current cognitive state in part by inhibiting oscillations in other frequencies (Engel and Fries, 2010). Given evidence that associative learning is accomplished through the synchronous firing of different populations of neurons at gamma frequency (Gruber et al., 2001), this may explain why elevated baseline beta activity is associated with poorer performance on associative learning and memory (PAL).

#### 4.1.3 Theta and delta oscillations

We found that theta oscillations were associated with better performance on PAL, and theta and delta oscillations were associated with better IED performance. These findings are somewhat surprising, given that resting theta power has been associated with poorer cognitive performance (see Klimesch, 1999 for review), while increased delta oscillations at rest are commonly considered to indicate brain pathology and are observed in a number of neurological and psychiatric conditions including schizophrenia, ADHD, Alzheimer’s disease, Parkinson’s disease, Down syndrome, depression, anxiety, and OCD (see Knyazev, 2012 for review). Delta and theta power also increase with normal aging (see Rossini et al., 2007 for review). However, age-dependent changes in the relationship between cognition and resting oscillations could offer a possible interpretation of the current findings. Several studies have found that enhanced delta and theta power show a positive relationship with cognitive performance in older, but not younger, adults (Finnigan and Robertson, 2011, Vlahou et al., 2014). It is possible that while increases in delta and theta power are observed in aging and a range of pathological conditions, this serves a compensatory function when faster oscillations (particularly alpha) are compromised. In this sense, patients with various psychiatric disorders may have a general pathology that results in increased delta and theta oscillations, but these oscillations may paradoxically preserve cognitive function.

### 4.2 Cognitive function across disorders

Performance on PAL, CRT, and IED did not differ significantly among diagnostic groups. This may suggest that executive function, episodic memory, and processing speed reflect transdiagnostic factors that are broadly affected across diagnostic categories. This is supported by a recent review of *meta*-analyses of neurocognitive impairment in psychiatric disorders, which found that deficits in executive function and episodic memory are the most severe and most frequently reported across disorders (East-Richard et al., 2019; MDD, schizophrenia, ASD, ADHD, bipolar, OCD, and PTSD were considered in the review). The authors suggest that deficits in executive function and episodic memory constitute key transdiagnostic neurocognitive impairments and may reflect common pathophysiological mechanisms across disorders. They suggest that a common cognitive factor may underlie various cognitive deficits across the diagnostic spectrum, akin to the “p factor” proposed by Caspi et al. (2014) that reflects an overall susceptibility to psychopathology. An impaired common cognitive factor could produce different cognitive deficits in different psychiatric disorders, and certain domains such as executive function and episodic memory may be more centrally related to a common cognitive factor and thus more often impaired. While processing speed was not identified as a central neurocognitive impairment, well-established associations between processing speed and oscillatory frequency (particularly alpha) suggest there may be a direct relationship between the speed of oscillatory and cognitive processes (Klimesch et al., 1997, Surwillo, 1963, Surwillo, 1964). This may explain why random forest models in the current study successfully identified neuro-oscillatory correlates of executive function, episodic memory, and processing speed, but not other cognitive domains. Deficits that are less severe or less ubiquitous across disorders may have simply been too slight to identify biological correlates.

The concept of a common cognitive factor also implies that cognitive dysfunction is a core mechanism in the pathophysiology of psychiatric illness, rather than a peripheral symptom. Cognition depends on the precise orchestration of cerebral activity, and cognitive dysfunction may therefore be the most direct and immediate consequence of pathophysiological alterations in cerebral networks in psychiatric disorders. It is therefore not surprising that EEG activity would be more strongly associated with cognitive function than with other symptoms or with disorders as a whole.

### 4.3 Limitations

The aim of the Across study is to examine dimensions of functioning without the categorical distinction between healthy, ‘normal’ control subjects and ill psychiatric patients. There is a broad range in cognitive functioning both in patient populations and among individuals without psychiatric diagnoses and this categorical distinction is a theoretical assumption that does not reflect clinical reality. However, since individuals without psychiatric diagnoses were not included in the current study it is unclear if our results reflect a general link between resting-state oscillations and cognitive function, or if our findings are specific to patients with psychiatric diagnoses and reflect a transdiagnostic pathological mechanism (i.e. biological correlates of cognitive *dys*function).

As such, we suggest that future transdiagnostic studies would benefit from the inclusion of healthy individuals not as comparison subjects per se, but to examine the full range of variability in cognition and other dimensions of functioning.

Finally, as is often the case in psychiatric research, medication status may influence the results. While this is mainly problematic in the comparison of individuals with psychiatric diagnoses to unmedicated controls, medication status may still introduce confounding effects that are difficult to control for given the diverse range of medications used in a transdiagnostic sample.

### 4.4 Clinical significance and implications

Resting state EEG is a widely used tool in psychiatric research, with a considerable number of studies linking specific disorders to power changes in specific frequency bands. Our results show that resting EEG oscillations are predictive of cognitive performance across several domains, but EEG data could not be used to predict diagnosis at a level above chance. Researchers should therefore be cautious when interpreting power abnormalities associated with psychiatric disorders. Without controlling for cognitive variables, it is possible that group differences in EEG frequency bands are driven by specific factors such as cognitive function that are not necessarily disorder specific.

While this may present a challenge to psychiatry’s search for disorder-specific EEG biomarkers, it could compel a transition to new approaches that link EEG features to more basic dimensions of functioning. For example, anhedonia has been considered a candidate endophenotype of depression and schizophrenia and is strongly linked to reduced electrophysiological responsiveness to reward (Padrao et al., 2013, Pizzagalli, 2014). We may develop a more biologically grounded understanding of (transdiagnostic) psychiatric illness by isolating cognitive, emotional, or social processes and linking these processes to specific neurophysiological correlates. By taking a fine-grained, bottom-up approach to link dimensions of psychiatric illness with biological parameters, we may discover novel patterns and identify new targets for treatment.

The current results could implicate resting-state oscillations as a potential treatment target to improve cognition for patients with severe deficits. In fact, existing research shows that neurofeedback training to increase resting upper alpha power can improve cognitive performance (Hanslmayr et al., 2005). Given the severe impact of cognitive impairment on general functioning and overall wellbeing, researchers and clinicians are calling for increased attention to cognitive deficits in psychiatric disorders. Some have suggested that efforts should be made to develop pharmacological therapies that specifically target cognitive deficits in psychiatric disorders (Etkin et al., 2013). Efforts to identify the neurobiological correlates of cognitive function may be the first step in the endeavor to develop such therapies and improve quality of life for patients.

**Funding/Support:** The author(s) received no specific external funding for this work. The study is supported through departmental resources. Dr. Verweij is supported by the Foundation Volksbond Rotterdam.

**Data Sharing Statement:** The data that support the findings of this study are available upon request at across@amsterdamumc.nl. The data requests will be discussed in the Across Executive board. The data are not publicly available due to the clinical and confidential nature of the data.
