# Supplementary material for: Resting-state brain oscillations predict cognitive function in psychiatric disorders: A transdiagnostic machine learning approach
Source: Neuroimage Clin. 2021 Mar 19;30:102617. doi: 10.1016/j.nicl.2021.102617 (PMC7985402; doi:10.1016/j.nicl.2021.102617)
Supplement: Supplementary Data 1 [file mmc1.docx]

**Supplementary Material**


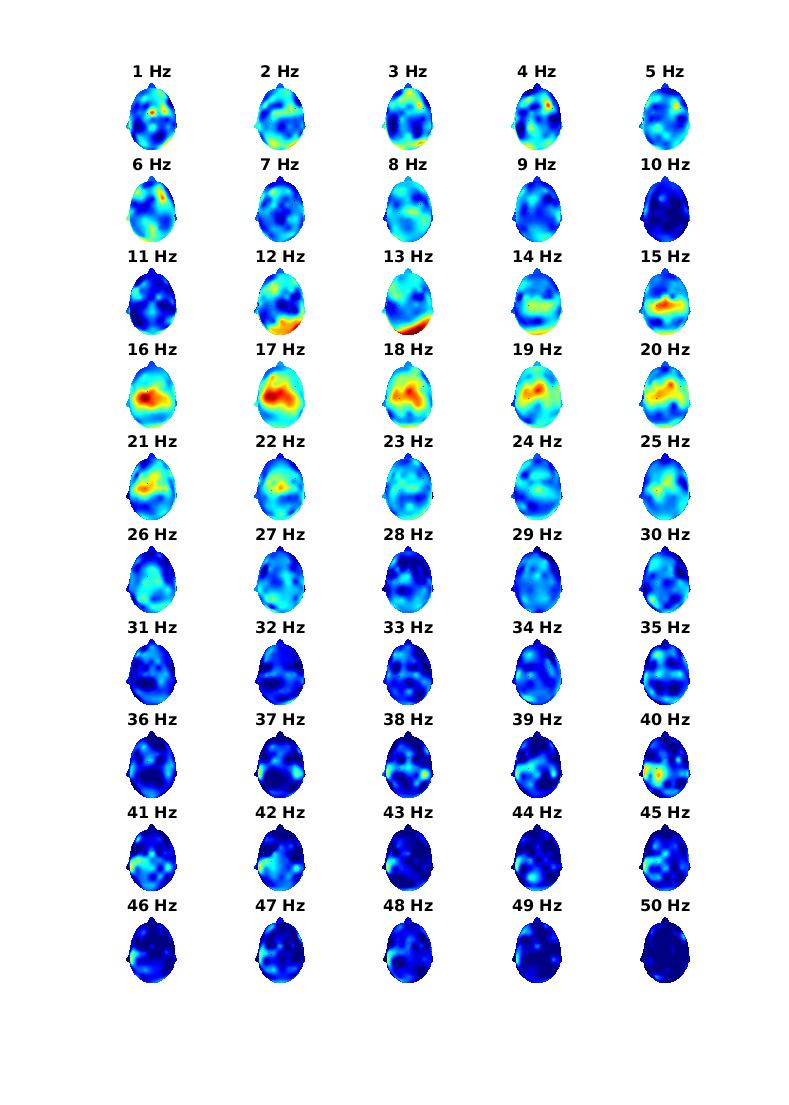


**Supplementary Figure 1**: Topography of PAL predictor importance at each frequency (1-50 Hz) in 1 Hz bins


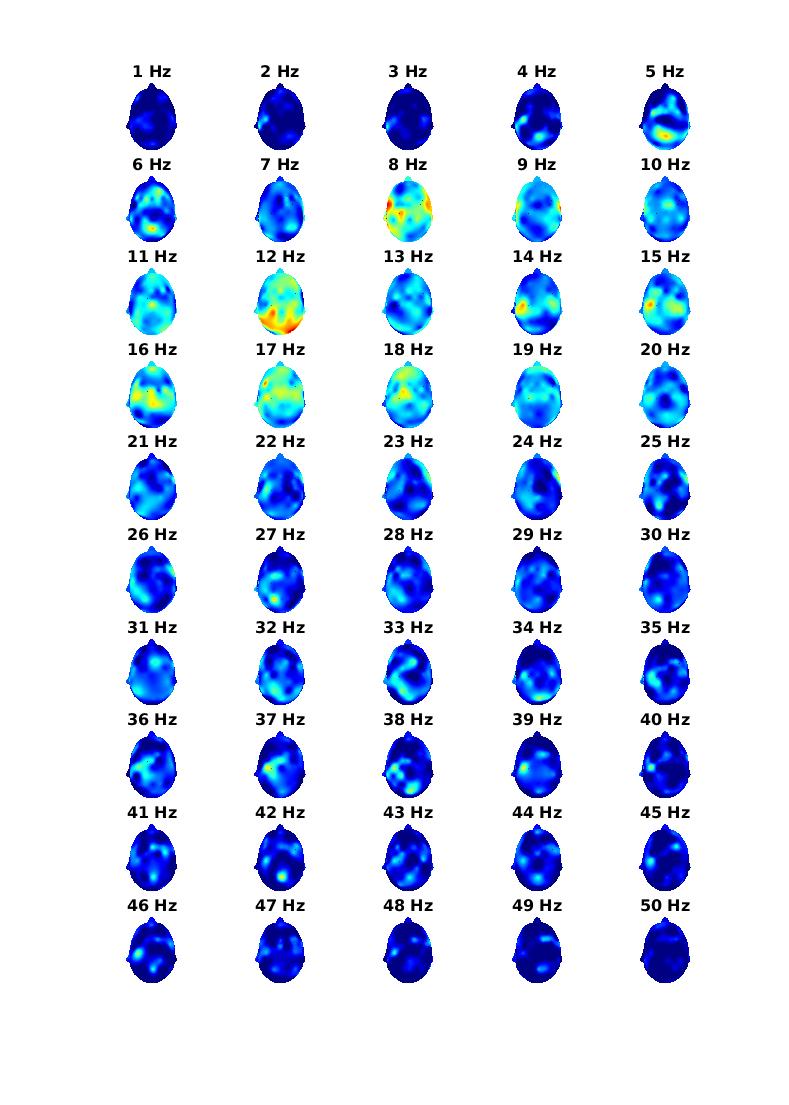


**Supplementary Figure 2**: Topography of CRT predictor importance at each frequency (1-50 Hz) in 1 Hz bins


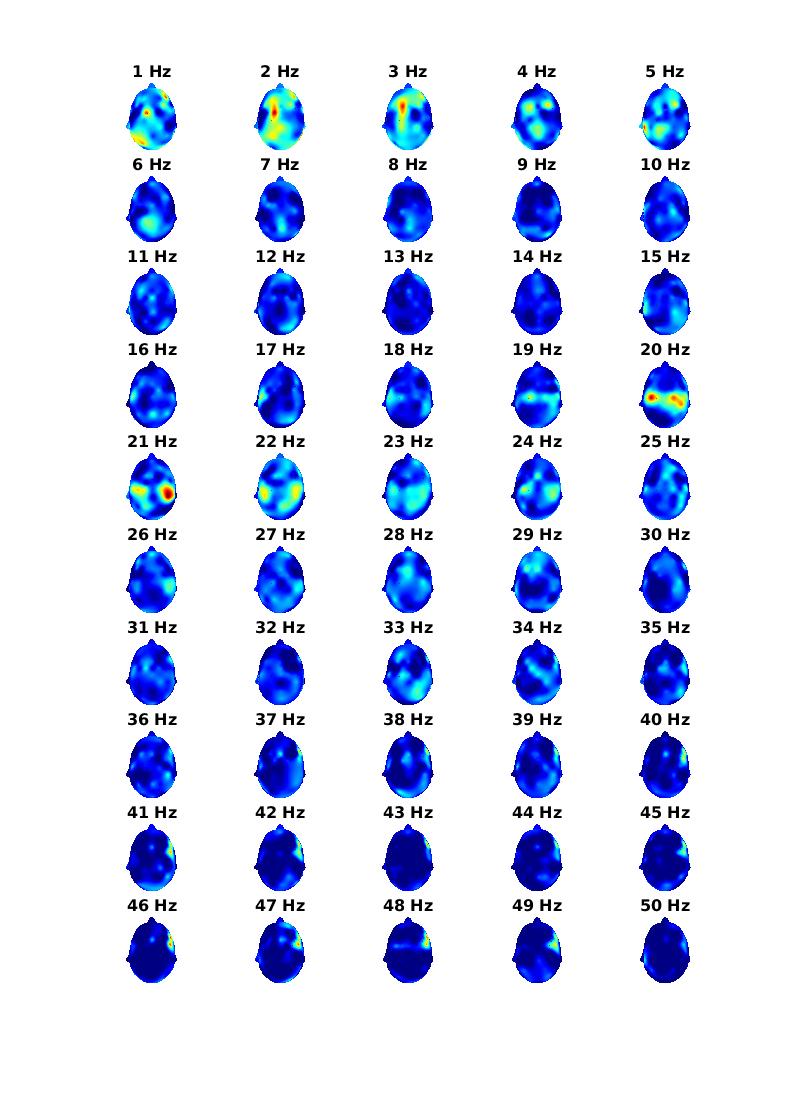


**Supplementary Figure 3**: Topography of IED predictor importance at each frequency (1-50 Hz) in 1 Hz bins

**Supplementary Table 1.** Medications and dosages

| **Medication type** | **Total** | | **MDD** | | **BP** | | **PSY** | | **OCD** | | **GAD** | | **ASD** | | **ID-NOS** | |
| --- | --- | --- | --- | --- | --- | --- | --- | --- | --- | --- | --- | --- | --- | --- | --- | --- |
|  | **Number** (%) | **Dosage (mg)** mean (SD) | **Number** (%) | **Dosage (mg)** mean (SD) | **Number** (%) | **Dosage (mg)** mean (SD) | **Number** (%) | **Dosage (mg)** mean (SD) | **Number** (%) | **Dosage (mg)** mean (SD) | **Number** (%) | **Dosage (mg)** mean (SD) | **Number** (%) | **Dosage (mg)** mean (SD) | **Number** (%) | **Dosage (mg)** mean (SD) |
| **SSRIs** | 37 (17.1) | 73.5 (72.1) | 9 (26.5) | 72.2 (52.1) | _ | _ | _ | _ | 8 (16.3) | 70.0 (69.1) | 4 (44.4) | 62.5 (61.8) | _ | _ | 16 (15.8) | 81.3 (91.0) |
| **TCAs** | 3 (1.4) | 158.3 (62.9) | 1 (2.9) | 100.0 (0.0) | _ | _ | _ | _ | 1 (2.0) | 150.0 (0.0) | _ | _ | _ | _ | 1 (1.0) | 225.0 (0.0) |
| **MAOIs** | 1 (0.5) | 70.0 (0.0) | _ | _ | _ | _ | _ | _ | _ | _ | _ | _ | _ | _ | 1 (1.0) | 70.0 (0.0) |
| **Typical antipsychotics** | 3 (1.4) | 2.7 (1.5) | 1 (2.9) | 3.0 (0.0) | _ | _ | 1 (6.6) | 4.0 (0.0) | 1 (2.0) | 1.0 (0.0) | _ | _ | _ | _ | _ | _ |
| **Atypical antipsychotics** | 14 (6.5) | 156.2 (227.0) | 1 (2.9) | 400.0 (0.0) | 1 (25.0) | 10.0 (0.0) | 8 (53.3) | 196.5 (268.0) | 3 (6.0) | 67.7 (114.6) | _ | _ | _ | _ | 1 (1.0) | 2.0 (0.0) |
| **Benzodiazepines** | 6 (2.8) | 7.8 (7.2) | 1 (2.9) | 1.0 (0.0) | _ | _ | _ | _ | 1 (2.0) | 20.0 (0.0) | _ | _ | _ | _ | 4 (4.0) | 6.5 (4.4) |
| **Psychostimulants** | 2 (0.9) | 25.0 (21.2) | 1 (2.9) | 10.0 (0.0) | _ | _ | _ | _ | _ | _ | _ | _ | _ | _ | 1 (1.0) | 40.0 (0.0) |
| **Anticonvulsants** | 2 (0.9) | 450.0 (212.1) | 2 (2.9) | 450.0 (212.1) | _ | _ | _ | _ | _ | _ | _ | _ | _ | _ | _ | _ |
| **All medications** | 68 (31.5) | 93.7 (138.3) | 16 (47.1) | 129.0 (167.8) | 1 (25.0) | 10.0 (0.0) | 9 (60.0) | 175.1 (258.7) | 14 (28.6) | 66.7 (75.1) | 4 (44.4) | 62.5 (61.8) | _ | _ | 24 (23.8) | 69.3 (86.5) |

*Note:* MDD = Major depressive disorder; BP = Bipolar disorder; PSY = Psychosis spectrum disorders; OCD = Obsessive-compulsive disorder; GAD = Generalized anxiety disorder; ASD = Autism spectrum disorder; ID-NOS = Impulse-control disorder, not otherwise specified (misophonia).SSRIs = selective serotonin reuptake inhibitors; TCAs = tricyclic antidepressants; MAOIs = monoamine oxidase inhibitors

**Medication Effects**

We investigated whether medication effects influenced relationships between EEG predictors and cognitive outcomes. This was to ensure that random forest regression results were not driven by stronger predictor-outcome relationships within a medicated (or unmedicated) subset of the sample. We selected EEG predictors by selecting each frequency peak identified for each cognitive test and calculating average power across the top three channels with highest predictor importance. This resulted in three EEG predictors per cognitive test. We then performed an ANCOVA for each EEG predictor to test for slope differences in predictor-outcome relationships among unmedicated patients (n=148), patients taking SSRIs (n=37), and patients taking atypical antipsychotics (n=14). Other medication classes were not included in the analysis due to small sample sizes. Slopes did not differ significantly among groups for any of the predictor-outcome effects. Supplementary Table 2 shows ANCOVA results.

**Supplementary Table 2.** ANCOVA Test of predictor-outcome slope differences among medication classes

|  |  | **Unmedicated** | **SSRIs** | **Antipsychotics** |  |  |
| --- | --- | --- | --- | --- | --- | --- |
|  |  | β (SE) | β (SE) | β (SE) | **F**(2,210) | **p-value** |
|  | **6 Hz** | -0.64 (0.34) | 0.37 (0.45) | -0.13 (0.51) | 1.45 | 0.24 |
| **PAL** | **13 Hz** | -0.46 (0.18) | 0.20 (0.23) | -0.02 (0.27) | 2.54 | 0.08 |
|  | **17 Hz** | 0.24 (0.32) | 1.16 (0.39) | 0.72 (0.55) | 1.75 | 0.18 |
|  |  |  |  |  |  |  |
|  | **8 Hz** | 0.31 (0.29) | 0.67 (0.33) | -0.36 (0.49) | 0.96 | 0.38 |
| **CRT** | **12 Hz** | -0.14 (0.19) | -0.21 (0.23) | -0.65 (0.30) | 0.66 | 0.52 |
|  | **17 Hz** | 0.37 (0.39) | 0.99 (0.46) | 1.24 (0.69) | 0.99 | 0.37 |
|  |  |  |  |  |  |  |
|  | **2 Hz** | -0.36 (0.40) | -1.35 (0.50) | -1.02 (0.64) | 1.34 | 0.27 |
| **IED** | **5 Hz** | -0.39 (0.39) | -1.04 (0.50) | -0.52 (0.59) | 0.46 | 0.63 |
|  | **22 Hz** | 0.07 (0.35) | 1.11 (0.42) | 0.81 (0.57) | 2.81 | 0.06 |

**Effects of diagnosis on predictor-outcome relationships**

Due to the large percentage of ID-NOS patients in the sample, we investigated whether relationships between EEG predictors and cognitive outcomes differed between ID-NOS patients and the rest of the sample. This was to ensure that random forest regression results were not driven by stronger predictor-outcome relationships within a subset of the sample. Using the same EEG predictors described above, we performed an ANCOVA for each EEG predictor to test for slope differences in predictor-outcome relationships between ID-NOS and non-ID-NOS patients. Slopes did not differ significantly between ID-NOS and non-ID-NOS patients for any of the predictor-outcome effects. Supplementary Table 3 shows ANCOVA results.

**Supplementary Table 3**. ANCOVA test of predictor-outcome slope differences between ID-NOS and non-ID-NOS patients

|  |  | **ID-NOS** | **Other diagnoses** |  | |
| --- | --- | --- | --- | --- | --- |
|  |  | β (SE) | β (SE) | **F** (1, 212) | **p-value** |
|  | **6 Hz** | -0.59 (0.22) | -0.26 (0.22) | 0.60 | 0.44 |
| **PAL** | **13 Hz** | -0.24 (0.12) | -0.28 (0.12) | 0.02 | 0.88 |
|  | **17 Hz** | 0.45 (0.17) | 0.59 (0.17) | 0.16 | 0.69 |
|  |  |  |  |  |  |
|  | **8 Hz** | 0.01 (0.17) | 0.59 (0.17) | 3.01 | 0.08 |
| **CRT** | **12 Hz** | -0.15 (0.12) | -0.19 (0.12) | 0.03 | 0.87 |
|  | **17 Hz** | 0.31 (0.20) | 0.66 (0.20) | 0.78 | 0.38 |
|  |  |  |  |  |  |
|  | **2 Hz** | -0.28 (0.24) | -0.77 (0.24) | 1.02 | 0.31 |
| **IED** | **5 Hz** | -0.25 (0.24) | -0.66 (0.24) | 0.72 | 0.40 |
|  | **22 Hz** | 0.27 (0.20) | 0.21 (0.20) | 0.02 | 0.89 |

While we tested whether cognitive performance differed among diagnostic groups, three diagnostic groups (BP, ASD, and GAD) were not included in the analysis due to their small sample sizes. To ensure that random forest regression results were not driven by these smaller groups, which in some cases had more extreme cognitive scores, we repeated the random forest regression and permutation procedure with only larger diagnostic groups included. All models still performed significantly better than chance, with the significance threshold at p=.0167 (.05/3 to correct for number of tests): PAL: NSE = .033, p = .0060; CRT: NSE = .0151, p = .0155; IED: NSE = .018, p = .0135. While model performance decreased somewhat for all tests, this may be due in part to the lower sample size.

**Symptom Dimensions**

**1. Objective**

Questionnaires on psychiatric symptoms often capture various underlying components of a psychiatric disorder and symptoms can cut across disorders, meaning that using sum-scores be inefficient as they can lead to a loss of information, such as on heterogeneity (i.e., two individual with the same score can endorse different symptoms). Furthermore, a questionnaire on depressive symptoms, for instance, could also ask about anxiety symptoms, and vice versa, potentially inflating comorbidity. The objective of this analysis was to identify transdiagnostic symptom dimensions by investigating the underlying structure of individual items of self-report psychiatric symptom questionnaires using data from participants with psychiatric disorders. Performing a factor analysis on individual symptoms of questionnaires can group related symptoms into smaller transdiagnostic dimensions, which could potentially be more precise than using sum-scores of categorical disorders.

**2. Materials and methods**

**2.1 Sample**

955 participants with psychiatric disorders from the Across study (Nieman et al., 2020). 45.1% of the sample were men and the mean age was 34.86 years (SD= 14.36). Inclusion criteria were: age 14-75 years, ability to give informed consent, have a DSM-IV-TR or DSM-5 diagnosis, clinically stability, and fluency in Dutch. Exclusion criteria were: high risk of suicide, unstable medical disorder, premorbid IQ<70, history of seizure or clinically significant abnormality of the neurological system. Informed consent is obtained from patients and their parents if patients are underage. Patients are able to discontinue participation from the study at any time.

**2.2 Materials**

*Hamilton Anxiety Scale* (HAM-A; Hamilton, Schutte, & Malouff, 1976). The HAM-A assesses the severity of somatic, cognitive, and affective symptoms of anxiety with 13 items. It consists of two subscales, psychological anxiety and somatic anxiety, and demonstrates good interrater reliability.

*Social Interaction Anxiety Scale* (SIAS; Mattick & Clarke, 1998). The SIAS measures anxiety in social interactions and fear of scrutiny by others with the 20-item and demonstrates high levels of internal consistency (α= 0.94), test-retest reliability at 12 weeks (*r*=0.92), and sensitivity to change with treatment (Mattick & Clarke, 1998).

*Prodromal Questionnaire-16* (PQ-16; Ising et al., 2012). The occurrence and severity of At Risk Mental State symptoms for a first psychosis is assessed with the PQ-16. It consists of 2 items on negative symptoms, 5 items on unusual thought content/delusional ideas/paranoia, and 9 items on perceptual abnormalities/hallucinations. Cronbach’s alpha for the total score was 0.77 and all item-total correlations were at least 0.31 (Ising et al., 2012).

*Inventory of Depressive Symptomatology Self-Report* (IDS-SR 30; Rush et al., 1986). The IDS measures the severity of depressive symptoms pertaining to mood, cognition, suicidality, arousal, and sleep with 30 items. It demonstrates good internal consistency (α = 0.85) and is applicable for different types of depression (Rush, Gullion, Basco, Jarrett, & Trivedi, 1996).

*Hedonism Scale* (Rombouts & Van-Kuilenburg, 1988)*.* The Hedonism Scale consists of 21 items and measures the degree of pleasure from physical activity, hearing, seeing, touching, tasting, sex and smelling.

**2.3 Statistical analysis**

An exploratory factor analysis (EFA) was conducted for data reduction using the *psych* package version 1.7.8 (Revelle, 2017) of the R statistical program (R Core Team, 2020). A correlation matrix was first created and tested with the Kaiser-Meyer-Olkin (KMO) test and Bartlett’s Test of Sphericity to determine the suitability of the items and data for EFA. Principal axis factoring was used as the extraction method due to the significantly non-normal distribution of the items (Costello & Osborne, 2005; Fabrigar, Wegener, MacCallum, & Strahan, 1999; Matsunaga, 2010). The number of factors retained was determined with parallel analysis, and oblique rotation was used to allow for correlation among factors (Costello & Osborne, 2005; Matsunaga, 2010) as it is reasonable to expect this. The cut-off score for each item factor loading was 0.30 (Costello & Osborne, 2005). Items with a communality lower than 0.3 were removed. From the parallel analysis, factor reduction was determined by the absence of Heywood cases (i.e., in which the communality is greater than or equal to 1) and a minimum of three items per factor.

**3. Results**

As per the KMO test, the overall Measure of Sampling Adequacy (MSA) for the correlation matrix of 46 items was 0.96, which is deemed adequate (Netemeyer, Bearden, & Sharma, 2003; Tabachnick & Fidell, 2001). Furthermore, the Bartlett’s Test of Sphericity was significant, demonstrating the suitability of the sample for analysis.

The parallel analysis identified 7 factors, which was reduce to 5, labelled: Social/Interpersonal, Depressive, Somatic, Anxious, and Anomalous. The factor loadings are shown in Table 1.

**Supplementary Table 4.** Factor Loadings

| **Item** | **Social/ Interpersonal** | **Depressive** | **Somatic** | **Anxious** | **Anomalous** |
| --- | --- | --- | --- | --- | --- |
| I felt uninterested in the things I used to enjoy (PQ-16) | 0.02 | **0.68** | 0.01 | -0.07 | 0.11 |
| I get extremely worried when I first meet people (PQ-16) | **0.53** | 0.04 | 0.00 | 0.05 | 0.21 |
| I have seen things that other people apparently  can't see (PQ-16) | -0.01 | 0.08 | 0.01 | -0.03 | **0.60** |
| I have heard things other people can't hear like  voices of people whispering or talking (PQ-16) | 0.06 | 0.06 | 0.04 | -0.06 | **0.55** |
| I have had the sense that some person or force is  around me, even though I could not see anyone (PQ-16) | 0.07 | 0.06 | 0.05 | 0.03 | **0.55** |
| Feeling Sad (IDS-SR) | 0.07 | **0.61** | -0.04 | 0.28 | -0.05 |
| Feeling Anxious or Tense (IDS-SR) | 0.11 | 0.16 | 0.09 | **0.59** | -0.07 |
| Response of Your Mood to Good or Desired Events (IDS-SR) | 0.02 | **0.60** | -0.04 | 0.15 | 0.07 |
| Concentration/Decision Making (IDS-SR) | 0.10 | **0.45** | 0.06 | 0.15 | -0.04 |
| View of Myself (IDS-SR) | 0.23 | **0.41** | -0.05 | 0.18 | -0.08 |
| View of My Future (IDS-SR) | 0.10 | **0.60** | 0.03 | 0.09 | -0.11 |
| Thoughts of Death or Suicide (IDS-SR) | 0.08 | **0.57** | -0.01 | 0.08 | -0.01 |
| General Interest (IDS-SR) | -0.01 | **0.77** | 0.05 | -0.01 | 0.09 |
| Energy Level (IDS-SR) | 0.03 | **0.45** | 0.27 | 0.08 | 0.04 |
| Capacity for Pleasure or Enjoyment (excluding sex) (IDS-SR) | -0.01 | **0.75** | 0.08 | 0.03 | 0.09 |
| Interest in Sex (IDS-SR) | 0.05 | **0.55** | 0.09 | -0.06 | -0.06 |
| Aches and pains (IDS-SR) | 0.03 | 0.05 | **0.66** | -0.07 | -0.11 |
| Other bodily symptoms (IDS-SR) | 0.01 | -0.01 | **0.66** | 0.07 | 0.06 |
| Panic/Phobic symptoms (IDS-SR) | 0.00 | -0.06 | 0.15 | **0.62** | 0.04 |
| Interpersonal Sensitivity (IDS-SR) | **0.42** | 0.06 | 0.08 | 0.23 | -0.06 |
| I get nervous if I have to speak with someone in authority (SIAS) | **0.63** | -0.01 | 0.04 | 0.08 | -0.02 |
| I have difficulty making eye contact with others (SIAS) | **0.62** | 0.02 | 0.00 | 0.03 | 0.18 |
| I become tense if I have to talk about myself or my feelings (SIAS) | **0.58** | 0.08 | 0.04 | 0.03 | -0.06 |
| I find it difficult to mix comfortably with the people I work with (SIAS) | **0.61** | 0.09 | -0.03 | 0.12 | 0.05 |
| I find it easy to make friends my own age (SIAS) | **0.57** | 0.16 | 0.03 | -0.18 | -0.08 |
| I tense up if I meet an acquaintance in the street (SIAS) | **0.66** | 0.00 | -0.11 | 0.14 | 0.15 |
| When mixing socially, I am uncomfortable (SIAS) | **0.71** | 0.09 | 0.06 | -0.03 | 0.00 |
| I feel tense if I am alone with just one other person (SIAS) | **0.64** | 0.02 | -0.02 | 0.09 | 0.07 |
| I am at ease meeting people at parties, etc (SIAS) | **0.57** | 0.24 | 0.06 | -0.16 | -0.09 |
| I have difficulty talking with other people (SIAS) | **0.73** | 0.11 | 0.01 | -0.11 | 0.01 |
| I find myself worrying that I won’t know what to say in social situations (SIAS) | **0.75** | 0.01 | -0.01 | -0.02 | 0.00 |
| I am nervous mixing with people I don’t know well (SIAS) | **0.83** | -0.05 | 0.03 | -0.03 | -0.05 |
| When mixing in a group, I find myself worrying I will be ignored (SIAS) | **0.76** | -0.10 | 0.01 | 0.04 | -0.10 |
| I am tense mixing in a group (SIAS) | **0.82** | -0.03 | 0.06 | 0.03 | -0.04 |
| I am unsure whether to greet someone I know only slightly (SIAS) | **0.71** | -0.11 | -0.02 | 0.06 | 0.11 |
| Anxious mood (HAM-A) | 0.04 | 0.12 | 0.06 | **0.71** | 0.01 |
| Tension (HAM-A) | 0.07 | 0.22 | 0.11 | **0.59** | 0.00 |
| Fears (HAM-A) | 0.04 | 0.00 | 0.07 | **0.68** | 0.03 |
| Insomnia (HAM-A) | -0.04 | 0.21 | **0.46** | 0.03 | -0.02 |
| Depressed mood (HAM-A) | 0.06 | **0.50** | 0.04 | 0.37 | -0.07 |
| Somatic (muscular) (HAM-A) | 0.01 | 0.10 | **0.65** | -0.07 | -0.06 |
| Somatic (sensory) (HAM-A) | -0.01 | 0.00 | **0.65** | 0.06 | 0.09 |
| Cardiovascular symptoms (HAM-A) | 0.00 | -0.05 | **0.60** | 0.13 | 0.04 |
| Respiratory symptoms (HAM-A) | 0.09 | -0.12 | **0.56** | 0.09 | 0.10 |
| Gastrointestinal symptoms (HAM-A) | 0.06 | -0.10 | **0.48** | 0.14 | 0.01 |
| Hedonism (Hedonism Scale) | -0.04 | **-0.61** | 0.02 | 0.09 | 0.06 |
| SS loadings | 8.36 | 5.82 | 3.54 | 3.47 | 1.25 |
| Proportion variance | 0.18 | 0.13 | 0.08 | 0.08 | 0.03 |

**Note.** PQ-16 = Prodromal Questionnaire-16, IDS-SR= Inventory of Depressive Symptomatology Self-Report, SIAS= Social Interaction Anxiety Scale, HAM-A= Hamilton Anxiety Scale. Loadings above 0.3 are bolded.

**References**

Costello, A. B., & Osborne, J. W. (2005). Best practices in exploratory factor analysis: Four recommendations for getting the most from your analysis. *Practical assessment, research & evaluation, 10*(7), 1-9.

Fabrigar, L. R., Wegener, D. T., MacCallum, R. C., & Strahan, E. J. (1999). Evaluating the use of exploratory factor analysis in psychological research. *Psychological Methods, 4*(3), 272.

Hamilton, M., Schutte, N., & Malouff, J. (1976). Hamilton anxiety scale (HAMA). *Sourcebook of Adult Assessment: Applied Clinical Psychology*, 154-157.

Ising, H. K., Veling, W., Loewy, R. L., Rietveld, M. W., Rietdijk, J., Dragt, S., . . . Linszen, D. H. (2012). The validity of the 16-item version of the Prodromal Questionnaire (PQ-16) to screen for ultra high risk of developing psychosis in the general help-seeking population. *Schizophrenia Bulletin, 38*(6), 1288-1296. doi:https://doi.org/10.1093/schbul/sbs068

Matsunaga, M. (2010). How to factor-analyze your data right: do’s, don’ts, and how-to’s. *International Journal of Psychological Research, 3*(1), 97-110.

Mattick, R. P., & Clarke, J. C. (1998). Development and validation of measures of social phobia scrutiny fear and social interaction anxiety. *Behaviour Research and Therapy, 36*(4), 455-470.

Nieman, D. H., Chavez-Baldini, U., Vulink, N. C., Smit, D. J. A., van Wingen, G., de Koning, P., . . . Denys, D. (2020). Protocol Across study: longitudinal transdiagnostic cognitive functioning, psychiatric symptoms, and biological parameters in patients with a psychiatric disorder. *BMC Psychiatry, 20*(1), 212. doi:10.1186/s12888-020-02624-x

R Core Team. (2020). R: A Language and Environment for Statistical Computing. Vienna, Austria: R Foundation for Statistical Computing. Retrieved from https://www.R-project.org

Revelle, W. R. (2017). psych: Procedures for personality and psychological research. Northwestern University. Retrieved from https://CRAN.R-project.org/package=psych

Rombouts, R., & Van-Kuilenburg, C. J. (1988). Hedonie, de ontwikkeling van een vragenlijst [Development of a questionnaire designed to measure hedonism]. *Gedrag en Gezondheid, 16*, 117-123.

Rush, A. J., Giles, D. E., Schlesser, M. A., Fulton, C. L., Weissenburger, J., & Burns, C. (1986). The inventory for depressive symptomatology (IDS): preliminary findings. *Psychiatry Research, 18*(1), 65-87. doi:https://doi.org/10.1016/0165-1781(86)90060-0

Rush, A. J., Gullion, C. M., Basco, M. R., Jarrett, R. B., & Trivedi, M. H. (1996). The inventory of depressive symptomatology (IDS): psychometric properties. *Psychological Medicine, 26*(3), 477-486.
